# Adrenomedullin in rat follicles and corpora lutea: expression, functions and interaction with endothelin-1

**DOI:** 10.1186/1477-7827-9-111

**Published:** 2011-08-09

**Authors:** Lei Li, Wai-Sum O, Fai Tang

**Affiliations:** 1 Department of Physiology, Li Ka Shing Faculty of Medicine, The University of Hong Kong, Pokfulam, Hong Kong SAR, China; 2 Department of Anatomy, Li Ka Shing Faculty of Medicine, The University of Hong Kong, Pokfulam, Hong Kong SAR, China; 3 Centre of Reproduction, Development and Growth, Li Ka Shing Faculty of Medicine, The University of Hong Kong, Pokfulam, Hong Kong SAR, China; 4 Centre of Heart, Brain, Hormone and Healthy Aging, Li Ka Shing Faculty of Medicine, The University of Hong Kong, Pokfulam, Hong Kong SAR, China

## Abstract

**Background:**

Adrenomedullin (ADM), a novel vasorelaxant peptide, was found in human/rat ovaries. The present study investigated the interaction of ADM and endothelin-1 (ET-1) in follicles and newly formed corpora lutea (CL) and the actions of ADM on progesterone production in CL during pregnancy.

**Methods:**

The peptide and gene expression level of adrenomedullin in small antral follicles, large antral follicles and CL was studied by real-time RT-PCR and EIA. The effect of ADM treatment on oestradiol production in 5-day follicular culture and on progesterone production from CL of different pregnant stages was measured by EIA. The interaction of ADM and ET-1 in follicles and CL at their gene expression level was studied by real-time RT-PCR.

**Results:**

In the rat ovary, the gene expression of *Adm *increased during development from small antral follicles to large antral follicles and CL. In vitro treatment of preantral follicular culture for 5 days with ADM increased oestradiol production but did not affect follicular growth or ovulation rate. The regulation of progesterone production by ADM in CL in culture was pregnancy-stage dependent, inhibitory at early and late pregnancy but stimulatory at mid-pregnancy, which might contribute to the high progesterone production rate of the CL at mid-pregnancy. Moreover, the interaction between ADM and ET-1 at both the production and functional levels indicates that these two vasoactive peptides may form an important local, fine-tuning regulatory system together with LH and prolactin for progesterone production in rat CL.

**Conclusions:**

As the CL is the major source of progesterone production even after the formation of placenta in rats, ADM may be an important regulator in progesterone production to meet the requirement of pregnancy.

## Background

First discovered in human pheochromatocytoma tissue in 1993 [1], adrenomedullin (ADM) is highly conserved across species [2-5] and is widely expressed in various organs and tissues, including heart, kidney, lung, adrenal gland [6] and reproductive organs, such as the ovary [7-10], the uterus [7,11], the oviduct [12], the testis [13-16], the prostate [17,18], and the epididymis [19]. ADM belongs to the calcitonin family with a high sequence homology to calcitonin gene-related peptide (CGRP). ADM can bind to the CGRP receptor in several types of tissues [20,21], but specific ADM receptors that are insensitive to CGRP receptor antagonist have been identified [22]. McLatchie et al. [23] demonstrated that the combination of calcitonin receptor-like receptor (CRLR) and receptor activity-modifying protein (RAMP) isoforms determines the ligand selectivity for CGRP and ADM. Coexpression of CRLR with RAMP1, RAMP2 and RAMP3 produces a CGRP receptor, an ADM1 and ADM2 receptor respectively.

ADM and its mRNA were reported in the follicles and the corpora lutea (CL) of rat [7] and human [9] ovaries and the levels varied during the oestrous cycle [12] or menstrual cycle [10]. In human ovary, *Adm *mRNA levels were low in the mature follicle but increased in the CL of the mid-luteal phase and remained high in CL of early pregnancy [9]. Immunoreactive ADM is predominantly localized in granulosa lutein cells at the mid-luteal phase and at late luteal phase [10]. ADM was also detected in human follicular fluid [10,24,25], with a level higher than that in the plasma [24]. ADM enhances progesterone production in human granulosa cells [10] but suppresses eCG-stimulated progesterone release in newly-formed eCG-primed rat CL [12]. On the other hand, endothelin-1 (ET-1) is known to inhibit progesterone production in the rat CL [26]. The interactions of ADM and ET-1 have been demonstrated in vascular smooth muscle cells (VSMC) [27-29], the glomerular mesangial cells (MC) in the kidney [30-32], the zona glomerulosa cells (ZGC) of the adrenal gland [33], and Leydig cells [15] and Sertoli cells [16] of the rat testis. In both the Leydig and Sertoli cells, ADM decreases ET-1 level but ET-1 increases ADM level [15,16]. The present study investigated the interaction of ADM and ET-1 in follicles and newly-formed CL and the action of ADM on progesterone production in CL during pregnancy.

## Methods

### Superovulation

Sprague-Dawley rats were obtained from the Laboratory Animal Unit, LKS Faculty of Medicine, the University of Hong Kong. The rats were housed at a constant temperature, humidity, under a 12-hour light-dark cycle (dark period 07:00 to 19:00) and rat chow and water were available ad libitum. All procedures related to animal usage were approved by the Committee on the Use of Live Animals for Teaching and Research, the University of Hong Kong.

Superovulation was induced in 21-23 days old immature female rats by intraperitoneal (i.p.) injection with 30IU per rat equine chorionic gonadotropin (eCG) and 20IU per rat human chorionic gonadotropin (HCG). An eCG injection was administered at 3:00 p.m. followed by a HCG injection 48 h thereafter. Small antral follicles (SAF) with a diameter between 250 μm - 400 μm and large antral follicles (LAF) of a diameter over 900 μm were isolated at 24 and 48 h post-eCG treatment. Newly-formed CL was isolated 24 h after HCG treatment. All rats were sacrificed by an overdose of sodium pentobarbital (40 mg/100 g of rat, Alfasan, Woerden, Holland). Follicles and CL were isolated in ice-cold normal saline under a dissecting microscope using two 26G1/2 inch syringe needles. The mRNA levels of *Adm *and its receptor components - *Crlr*, *Ramp1*, *Ramp2 *and *Ramp3 *together with *Et1 *were studied by real-time RT-PCR. To study the tissue level of ADM peptide and its secretion, isolated small antral follicles, large antral follicles, and CL from 5 rats were pooled together and divided randomly into a 24-well plate at 9 small antral follicles, 3 large antral follicles or 3 CL per well. Follicles and CL were pre-incubated for 1 h in 0.5 ml DMEM/F-12 medium (Invitrogen life technologies, Carlsbad, CA, USA) and then incubated for another 6 h. The incubation was carried out at 37°C under 95% humidity and 5% CO_2_. At the end of the incubation, culture media and, follicles or CL was collected for ADM measurement.

### Effect of ADM on follicular development

Female 21-23 day old SD rats were sacrificed by an overdose of sodium pentobarbital (40 mg/100 g of rat, Alfasan, Woerden, Holland). Their ovaries were removed and transferred to a pre-warmed follicle isolation medium - Leibovitz's L-15 medium (Invitrogen life technologies, Carlsbad, CA, USA) with 0.1% BSA, pH 7.6. The ovaries were washed once with L-15 medium, cut into 2-3 pieces each and then transferred to an ovarian-tissue digestion medium (1 × αMEM with 2 mg/ml collagenase, 0.2 mg/ml DNase I; Gibco BRL, Grand Island, NY, USA). Digestion was carried out in a 37°C incubator for about 5 min and then stopped by washing the ovarian tissues with a pre-warmed L-15 medium. The ovarian tissues were further incubated in a fresh L-15 medium for 15-30 min in a 37°C incubator. Preantral follicles (diameter of 170-210 μm) were dissected out, washed for 3 times by 1 × DMEM/F-12 medium and transferred into 96-well plate, one for each well with 100 μl follicle culture medium (1 × αMEM with 5 μg/ml insulin, 10 μg/ml transferring, 25 μg/ml ascorbic acid, 1 ng/ml sodium selenite, 5 IU/ml FSH, 0.25 IU/ml LH, 5% FBS) and pre-incubated at 37°C for 3 h. Then the culture medium was replaced with a new follicle culture medium with or without 100 nM ADM. The follicular "diameter" (which is the average value of the length and width) was measured under an inverted microscope to make sure they were within 170 - 210 μm. This was recorded as day 0 measurement. The follicles were cultured in an inverted 96-well plate in a 37°C, 95% humidity and 5% CO_2 _incubator. Fresh follicle culture medium was changed every other day. The "diameter" of each follicle was measured on days 1, 2 and 4. The culture medium for incubation from day 2 to day 4 (48 h incubation) was collected and the oestradiol was assayed by an EIA kit.

### ADM and ET-1 interaction in follicles and CL and regulation of ADM by progesterone and HCG

The interaction of ADM and ET-1 in large antral follicles and newly-formed CL from superovulation model was studied. Follicles from 5 rats were pooled together and divided randomly into a 24-well plate at 3 follicles per well. They were pre-incubated for 1 h in 0.5 ml DMEM/F-12 medium. The medium was then replaced and receptor antagonists, 1 μM hADM_22-52 _or 1 μM hCGRP_8-37 _(Phoenix Pharmaceuticals, Burlingame, CA) was added into the culture medium and incubated for 1/2 h. After that 100 nM ADM (Phoenix Pharmaceuticals, Burlingame, CA) was added into the culture medium and incubated for another 6 h. Pre-incubated follicles were also treated with 100 nM ET-1 (Phoenix Pharmaceuticals, Burlingame, CA). All the incubations were carried out at 37°C under 95% humidity and 5% CO_2_. As to CL, CL from 6-8 rats were pooled together and divided randomly into a 24-well plate at 3 CL per well in 0.5 ml medium. The incubation procedure was the same as that of follicles. At the end of the incubation, culture media was collected for the measurement of ADM or ET-1 by enzyme immunoassay (EIA). Follicles/CL were harvested for protein measurement for the normalization of peptide production or for the study of the gene expression levels of *Adm *and *Et1 *and their respective receptor components.

To study the effects of progesterone and HCG, 10 nmol/L progesterone or 0.75 IU/ml HCG was added into the culture medium after 1 h pre-incubation. After 6 h incubation, the CL was harvested to determine the mRNA levels of *Adm *and its receptor components by real-time RT-PCR.

### Short-time incubation of CL from pregnant rats

Proestrus females were caged with proven fertile males overnight and the presence of vaginal sperm in the following morning was taken as evidence of successful copulation. This was day 1 of pregnancy. To study the effect of ADM on steroidogenesis, CL from ovaries of 7-day, 12-day and 17-day pregnant rats were isolated under a dissecting microscope with two 26G1/2 syringe needles in ice-cold normal saline. CL from 5-6 rats of each gestational age were dissected out, pooled and randomly divided into a 24-well plate at 3-4 CL per well in 0.5 ml DMEM/F-12 medium. First, the isolated CL was pre-incubated in 37°C for 1 h. The medium was replaced and a receptor antagonist, 1 μM hADM_22-52 _or 1 μM hCGRP_8-37_, was added into the culture medium and incubated for 1/2 h. After that 100 nM ADM was added into the culture medium. At the end of 6 h incubation, the culture medium was collected for the measurement of progesterone production by EIA. The CL was harvested for protein measurement by a Pierce BCA protein assay kit (Thermo Scientific, Rockford, IL, USA) for the normalization of the hormone production.

### Short-time incubation of placenta

The placentae of 12-day and 17-day pregnant rats were dissected out, and each was cut into 16 or 32 pieces in ice-cold normal saline. Three pieces of placenta tissue were randomly distributed in one well of a 24-well plate. The incubated procedure was the same as that for the short-time incubation of CL, except that the volume of the culture medium was 1.0 ml instead of 0.5 ml. The culture media and placenta tissues were collected for progesterone EIA and protein normalization by Pierce BCA protein assay respectively.

### Genes expression of *Adm*, *Crlr*, *Ramp1*, *Ramp2*, *Ramp3, Et1, Eta and Etb*

Total RNA of the follicles and corpus luteum were extracted using TRIZOL reagent and subjected to real-time RT-PCR analysis. RNA samples (1 μg) were reverse transcribed into cDNA with the iScript reverse transcriptase according to the manufacturer's instructions (Bio-Rad Laboratories, Hercules, CA). The real-time RT-PCR setup was previously described [12]. The reaction mixtures contained 25 μl iQ SYBR Green Supermix (Bio-Rad Laboratories, Hercules, CA), 500 nM of each primer, 1 μl template cDNA, and DNase-free water to a final volume of 50 μl. Cycle conditions were 95°C for 10 min, followed by 40 cycles of 95°C for 45 sec, 59°C for 30 sec, and 72°C for 45 sec. The reaction was completed with a dissociation step for melting point analysis from 50°C to 95°C (in increments of 0.5°C for 10 sec each. The primers were designed on the basis of the published sequences of *Adm *(caggacaagcagagcacgtc, forward; tctggcggtagcgtttgac, reverse); *Crlr *(ccaaacagacttgggagtcactagg, forward; gctgtcttctctttctcatgcgtgc, reverse); *Ramp1 *(cactcactgcaccaaactcgtg, forward; cagtcatgagcagtgtgaccgtaa, reverse); *Ramp2 *(aggtattacagcaacctgcggt, forward; acatcctctgggggatcggaga, reverse); *Ramp3 *(acctgtcggagttcatcgtg, forward; acttcatccggggggtcttc, reverse); *Et1*(tctcttgcctcttcttgctgtc, forward; gaaactccgccctgctatgg, reverse); *Eta *(ccgtctatgatgagatggataag, forward; ggttgccaggttaatgcc, reverse) *Etb *(ccgtgcgagactgaaaac, forward; ccgagaagagatggtgtgg, reverse) and *Actb *(ggaaatcgtgcgtgacatta, forward; aggaaggaaggctggaagag, reverse). Standard curves for each gene product were prepared using serial dilution of the cDNA to determine PCR efficiency. The PCR efficiencies for *Adm*, *Crlr*, *Ramp1*, *Ramp2*, *Ramp3*, *Et1, Eta *and *Etb *and *Actb *were all above 0.95 and similar. The relative gene expression levels normalized to *Actb *were analyzed using the ΔΔCT method, where CT was the cycle threshold. Melt curve analysis for each primer set revealed only one peak for each product. The size of the PCR products was confirmed by comparing the size of product with a commercial ladder after agarose gel electrophoresis.

### Extraction of peptides from culture medium

The peptides in the culture medium were extracted, purified and concentrated by reversed-phase extraction using Sep-Pak^® ^Vac C18 Cartridges (200 mg, Waters Corp., Milford, Massachusetts, USA). The peptides in the eluate were then concentrated by a Savant speed-vacuum concentrator (Savant, Farmingdale, NY, USA) for 2 h and freeze-dried in a lypholizer (Labconco Corp, Kansas City, MO, USA) overnight. The dried extract was re-constituted in 1 × EIA assay buffer. The peptide level in the culture medium was normalized by the amount of protein in the tissue as measured by the Pierce BCA protein assay kit.

### Extraction of peptides from follicles and CL

Follicles and corpora lutea were homogenized in 2 N ice-cold acetic acid and then boiled for 10 min. A 50 μl aliquot was taken for Bio-Rad protein assay (Bio-Rad Laboratories, Hercules, CA) for normalization. The remaining homogenates were centrifuged at 18, 600 × g for 20 min at 4°C. The supernatants were lyophilized and stored at -20°C until assayed for ADM immunoreactivity.

### Enzyme Immuno Assay (EIA)

An ADM 1-50 (rat) EIA kit (Phoenix Pharmaceuticals, Inc, Burlingame, CA, USA) was employed to measure ADM. The minimum detectable concentration is 0.15 ng/ml and the range is 0-100 ng/ml. The intra-assay error and inter-assay error were less than 5% and 14% respectively. ET-1 was measured with an ET-1 EIA kit (Phoenix Pharmaceuticals, Inc, Burlingame, CA, USA). The minimum detectable concentration is 0.1 ng/ml and the range is 0-25 ng/ml. The intra-assay error and inter-assay error were less than 5-10% and 15% respectively.

The progesterone EIA kit (MP Biomedicals, NY) has a minimum detectable concentration of 0.3 ng/ml and a range of 0-50 ng/ml. The intra-assay error and inter-assay error for this progesterone EIA kit is 2.4% and 2.6% respectively. The oestradiol EIA kit (MP Biomedicals, NY) has a minimum detectable concentration of 10 pg/ml and a range of 0-1000 pg/ml. The intra-assay error and inter-assay error for oestradiol EIA kit is 4.9% and 6.6% respectively.

### Statistical analysis

All data were expressed as mean ± SEM, and statistical significance was assessed by one-way ANOVA followed by Fisher's least significant difference test for post-hoc comparisons. Statistical significance was taken at the P < 0.05 level.

## Results

### ADM and gene expression of *Adm *and its receptor components in follicles and CL

**Figure 1:**
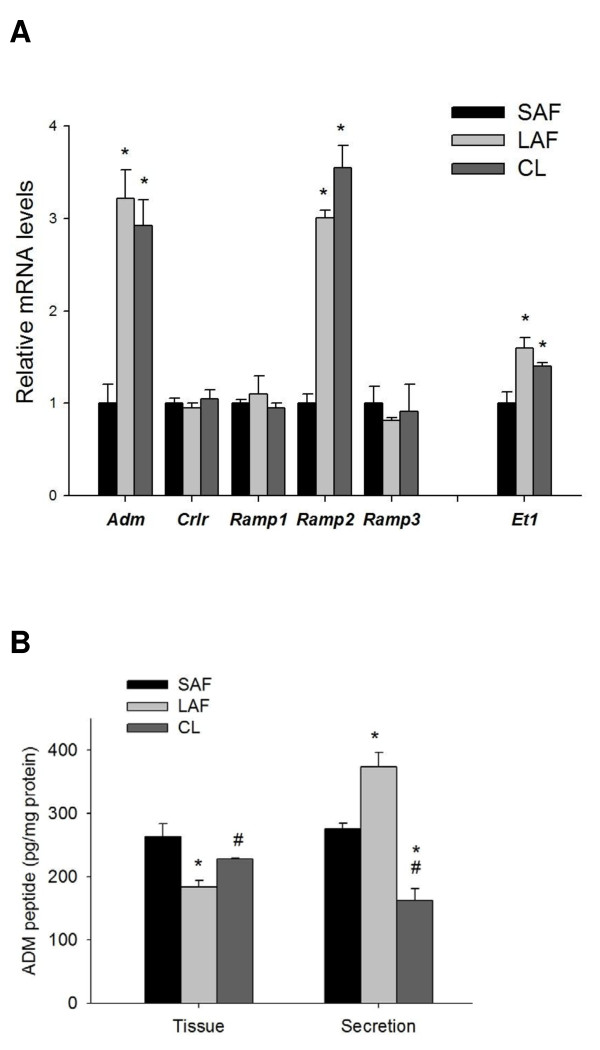
**The peptide and gene expression levels of adrenomedullin in follicles and corpus luteum (CL)**. The gene expression levels of *Adm*, its receptor components and *Et1 *in small antral follicles (SAF), large antral follicles (LAF) and newly-formed corpus luteum (CL) (A); tissue levels of ADM and secreted ADM levels from SAF, LAF and CL after 6 h incubation (B). n = 5. * P < 0.05 compared with SAF; #P < 0.05 compared with LAF.

The gene expression level of *Adm *and *Ramp2 *in large antral follicles and CL were much higher than those in small antral follicles (Figure 1A), while the expression levels of *Crlr*, *Ramp1 *and *Ramp3 *showed no significant difference (Figure 1A). There was no significant difference in the gene expression levels in either *Adm *or its receptor components between LAF and CL. After 6 h incubation, large antral follicles secreted more, and CL secreted less, ADM into the medium than small antral follicles (Figure 1B). The tissue levels of ADM peptide were lower in large antral follicles and CL compared with small antral follicles (Figure 1B).

### Interaction of ADM and ET-1 in follicles

**Figure 2:**
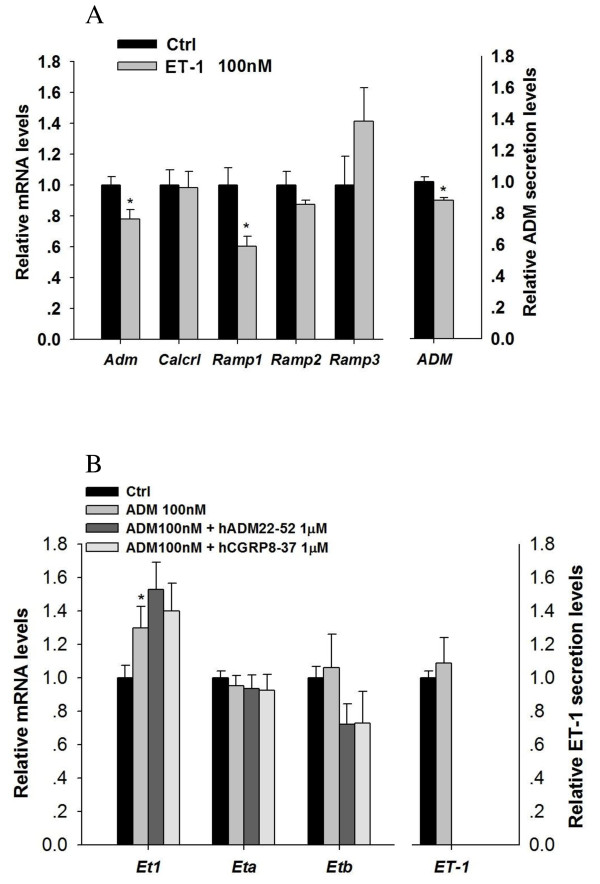
**Interactions of ADM and ET-1 in large antral follicles**. ET-1 reduced the gene expression levels of *Adm *and *Ramp1*, and the ADM peptide secretion level (A); ADM treatment up-regulated *Et1 *gene expression level (B). Data were presented as mean ± SEM. n = 5; *P < 0.05 compared with control.

After 6 h incubation, ET-1 reduced ADM secretion and the mRNA levels of *Adm *and *Ramp1*, but not *Crlr*, *Ramp2 *and *Ramp3*, in the large antral follicle (Figure 2A). On the contrary, ADM treatment up-regulated *Et1 *mRNA levels without affecting those of *Eta *and *Etb *receptors and ET-1 secretion (Figure 2B). The stimulatory effect of ADM on *Et1 *expression could not be abolished by either hADM_22-52 _(an ADM receptor antagonist) or hCGRP_8-37 _(a CGRP receptor antagonist) (Figure 2B).

### Interaction of ADM and ET-1 in CL and regulation of ADM expression by progesterone and HCG

**Figure 3:**
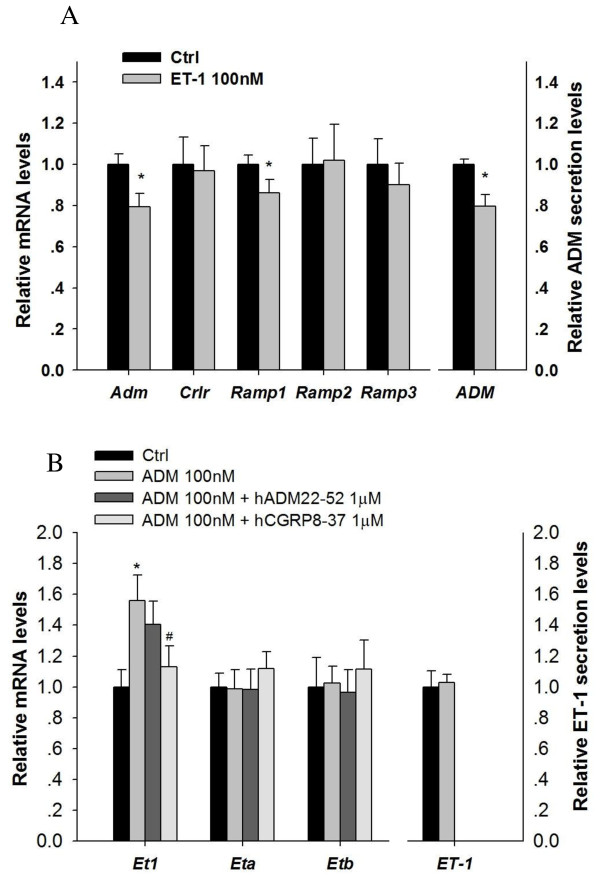
**Interactions of ADM and ET-1 in corpus luteum (CL)**. ET-1 reduced *Adm *and *Ramp1 *gene expression and ADM secretion in the CL (A); ADM treatment increased *Et1 *expression level, which was reversed by a CGRP receptor blocker - hCGRP_8-37 _(B). n = 6-8; *P < 0.05 compared with control, #P < 0.05 compared with 100 nM ADM treatment group.

**Figure 4:**
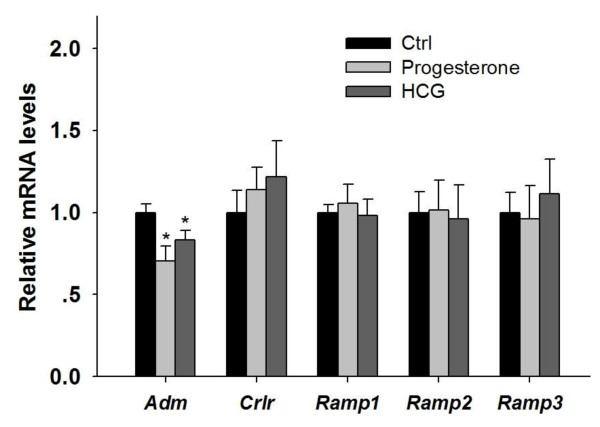
**Regulation of gene expression of *Adm *and its receptor components in the corpus luteum (CL) by progesterone and HCG**. Progesterone and HCG both suppressed *Adm *expression. n = 5; *P < 0.05 compared with control.

ET-1 reduced *Adm *gene expression and ADM secretion in the CL after 6 h incubation (Figure 3A) while ADM treatment increased *Et1 *expression level, an effect which was abolished by a CGRP receptor blocker - hCGRP_8-37 _(Figure 3B). Both progesterone and HCG suppressed *Adm *expression but had no significant effect on the expression levels of *Crlr*, *Ramp1*, *Ramp2 *or *Ramp3 *(Figure 4).

### Effect of ADM on follicular growth and oestradiol secretion

The cultured follicles were examined daily under microscopy. Most of the cultured preantral follicles increased in size and their diameter increased from 185.9 ± 3.1 μm to 347.6 ± 18.0 μm after 96 h incubation. There was no significant difference in follicular size between the control and ADM-treated group after 24 h, 48 h and 96 h incubation. However, oestradiol secretion into the culture medium of ADM-treated group (960.8 ± 136.1 pg/follicle) was significantly higher than the control group (600.5 ± 98.8 pg/follicle).

### Effect of ADM on progesterone production from CL and placenta of pregnancy

**Figure 5:**
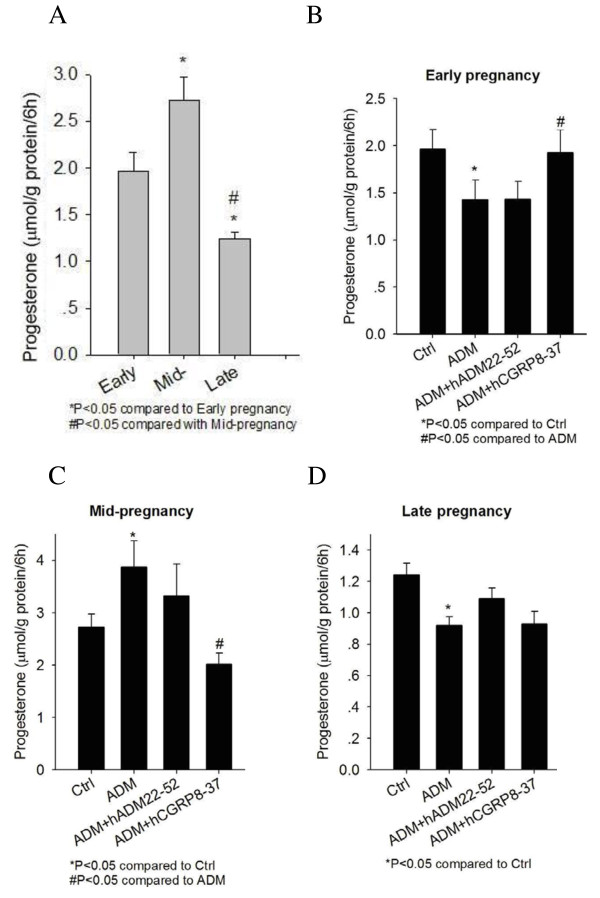
**Progesterone production from corpus luteum (CL) of early (7-day), mid- (12-day) and late (17-day) pregnant rats (A) and the effects of ADM treatment on progesterone production after 6 h incubation**. ADM suppressed progesterone production in CL from early (B) and late pregnancy (D), while increased progesterone production in CL from mid-pregnancy, which was diminished by co-treatment with hCGRP_8-37 _(C). Data were presented as mean ± SEM. n = 5-6.

As shown in Figure 5A, the basal rates of progesterone production from CL of early, mid- and late pregnant rats were different, with the highest progesterone production rate at mid-pregnancy. Treatment with 1 nM or 10 nM ADM had no effect on progesterone production (data not shown). However, 100 nM ADM showed different effects on progesterone production in CL at different stages of gestation - inhibitory in early pregnancy (Figure 5B) and late pregnancy (Figure 5D) but stimulatory in mid-pregnancy (Figure 5C). The inhibitory effect in early pregnancy and the stimulatory effect at mid-pregnancy were reversed by the CGRP receptor antagonist, hCGRP_8-37_.

The secretion level of progesterone from the placenta was extremely low compared with those in CL, with 11.4 ± 4.7 and 3.6 ± 0.2 nmol/g protein/6 h for placenta of mid- and late pregnancy respectively. A CL produced much more progesterone than a placenta (184.4 ± 50.2 and 208.9 ± 32.3 picomol per CL vs 19.4 ± 17.5 and 19.4 ± 2.7 picomol per placenta at mid- and late pregnancy). ADM had no effect on progesterone production in placentae (data not shown).

## Discussion

In the rat, the recruitment of follicles into the ovulatory cohort occurs around the time of antrum formation with a follicle diameter of about 200-400 μm, concomitant with the atresia of most of the follicles [34]. Ovulation occurs when the follicles reach a size of 900-1000 μm in diameter [34]. The finding of a higher ADM secretion and *Adm *mRNA level in large antral follicles and CL compared with small antral follicles agrees well with the previous qualitative description of increased ADM and *Adm *mRNA expression in the human CL [9], and suggests a possible role of ADM in follicle development, luteogenesis and/or steroidogenesis. High *Ramp2 *gene expression levels in large antral follicles and CL compared to small antral follicles suggests a possible further augmentation of ADM function through the increase in an ADM receptor.

After a short 6-h incubation, large antral follicles secreted the largest amount of ADM peptide into the medium. High ADM level in human follicular fluid has been reported and the pre-ovulatory level is even higher than the plasma ADM concentration [10,24,25]. The possible function of secreted ADM from the large antral follicles remained unknown. After ovulation the ADM in the residual follicular fluid that enters the oviduct together with the oocyte may exert biological actions in the fallopian tube and the uterus, such as the regulation of blood flow [35], sperm transportation [36,37] and uterine contraction [10].

ADM at the dose of 100 nM had no effects on the follicular size and follicular survival rate. In the heterozygous *Adm *knockout mouse with decreased *Adm *expression and reduced fertility, there was also no significant decrease in ovulation, fertilization rate or overt defects in folliculogenesis or CL formation [38]. On the other hand, ADM was found to stimulate oestradiol production from the follicles, suggesting a role for ADM in steroidogenesis rather than granulosa cell proliferation.

The relative high levels of ADM and the gene expression level of *Adm *and *Ramp2 *in the newly-formed CL also suggested that ADM might be involved in CL formation or luteal functions. Indeed our group has reported an inhibitory effect of ADM on eCG-stimulated progesterone secretion from CL [12]. The CL employed in that report were isolated from eCG primed rats, which were not fully functional as compared to the CL of pregnant rats. We therefore used CL from early, mid- and late pregnant rats for this study. It is of interest to note that ADM showed opposite effects on progesterone production depending on the pregnancy stage, inhibitory in CL from early and late pregnant rat while stimulatory in CL from mid-pregnant rats. This dual role of ADM in progesterone production may partly explain for the differences in the basal progesterone production from the CL at different pregnancy stages. CL of mid-pregnancy produced the greatest amount of ADM when the effect of ADM is stimulatory, not forgetting that the low basal progesterone secretion from the CL of late pregnancy could be due to the decrease in LH and prolactin secretion from early pregnancy onwards [39] while the increase in progesterone production in mid-pregnancy could be due to the secretion of placental luteotopin with prolactin activity from day 6 to day 11 of pregnancy [40]. Prolactin [41] and placental luteotropins [42] are known to stimulate luteal progesterone production

The difference in ADM effects on progesterone production cannot be simply due to the binding to different receptors as both the inhibitory effect in early pregnancy and the stimulatory effect in mid-pregnancy were blocked by the CGRP receptor antagonist. In late pregnancy, the inhibitory effect was more likely mediated by the ADM receptor. A similar dual effect has been reported for the actions of progesterone on prolactin secretion, with a stimulatory effect in early pregnancy (day 7) and an inhibitory effect in late pregnancy (day 17) [43]. The suppressive action of progesterone on *Adm *gene expression may suggest the presence of a negative feedback between ADM and progesterone. The inhibitory effect of hCG on CL *Adm *gene expression is in line with the FSH effect in the follicles [8].

Compared with CL, the progesterone production from placenta was minor. This is consistent with existing literature that the progesterone production in rat CL was not taken over by placenta from mid-pregnancy onwards, unlike the situation in human pregnancy [44]. In our study, progesterone production of the placenta decreased from mid-pregnancy to late pregnancy, in line with the finding of Matt and MacDonald [44].

It was found that in both the follicle and CL, ADM and ET-1 showed opposite regulatory effect on each other, indicating the presence of an autocrine/paracrine regulatory loop between ADM and ET-1. ADM stimulated the gene expression level of *Et1 *in the CL, an effect which is abolished by CGRP receptor blocker - hCGRP_8-37_. As ET-1 was reported to inhibit progesterone production in the rat CL [26], the stimulatory effect of ADM on *Et1 *gene expression might in turn suppress progesterone production. On the other hand, ET-1 treatment suppressed both the ADM peptide secretion and its gene expression in CL. This finding is opposite to the results in both the Leydig and Sertoli cells, in which ADM decreases ET-1 level but ET-1 increases ADM level [15,16]. It suggests that besides a direct inhibitory effect, ET-1 may also fine-tune progesterone production through its suppressive effects on ADM secretion and *Adm *gene expression. In addition to the functional interaction of ADM and ET-1 in regulating progesterone, in the CL, ADM may also have actions opposite to those of ET-1 in luteolysis. ET-1 is suggested to be luteolytic [26,45] while ADM may be anti-luteolytic [9] by maintaining adequate luteal blood flow as a vasodilator.

## Conclusions

In conclusion, the gene expression of *Adm *and ADM secretion increased with the change from small antral follicles to large antral follicles and CL. ADM in the follicle was involved in steroidogenesis. In CL of pregnancy, the regulation of ADM on progesterone production was pregnancy stage - dependent, inhibitory in CL from early and late pregnancy while stimulatory at mid-pregnancy. Moreover, there was the interaction between ADM and ET-1 at both the production and functional levels in CL. Besides prolactin and placental luteotropins, ADM may be an important regulator in progesterone production during pregnancy, being itself under the inhibitory effects of LH, progesterone and ET-1.

## Competing interests

The authors declare that they have no competing interests.

## Authors' contributions

LL worked on mating of rats, collection of tissues, in vitro culture of the tissues, real time PCR, ELISA and drafted the manuscript. WSO and FT coordinated the project. WSO and FT are respectively principal and co-investigators of the research and holders of the grant. Both WSO and FT helped in the revision of the manuscript. All authors read and approved the final manuscript.
